# The Role of Nursing Homes in the Spread of Antimicrobial Resistance Over the Healthcare Network

**DOI:** 10.1017/ice.2016.59

**Published:** 2016-04-07

**Authors:** Carline van den Dool, Anja Haenen, Tjalling Leenstra, Jacco Wallinga

**Affiliations:** 1 Center for Infectious Disease Control (CIb), National Institute for Public Health and the Environment (RIVM), Bilthoven, The Netherlands; 2 Department of Medical Statistics and Bioinformatics, Leiden University Medical Center, Leiden, The Netherlands

## Abstract

**OBJECTIVE:**

Recerntly, the role of the healthcare network, defined as a set of hospitals linked by patient transfers, has been increasingly considered in the control of antimicrobial resistance. Here, we investigate the potential impact of nursing homes on the spread of antimicrobial-resistant pathogens across the healthcare network and its importance for control strategies.

**METHODS:**

Based on patient transfer data, we designed a network model representing the Dutch healthcare system of hospitals and nursing homes. We simulated the spread of an antimicrobial-resistant pathogen across the healthcare network, and we modeled transmission within institutions using a stochastic susceptible–infected–susceptible (SIS) epidemic model. Transmission between institutions followed transfers. We identified the contribution of nursing homes to the dispersal of the pathogen by comparing simulations of the network with and without nursing homes.

**RESULTS:**

Our results strongly suggest that nursing homes in the Netherlands have the potential to drive and sustain epidemics across the healthcare network. Even when the daily probability of transmission in nursing homes is much lower than in hospitals, transmission of resistance can be more effective because of the much longer length of stay of patients in nursing homes.

**CONCLUSIONS:**

If an antimicrobial-resistant pathogen emerges that spreads easily within nursing homes, control efforts aimed at hospitals may no longer be effective in preventing nationwide outbreaks. It is important to consider nursing homes in planning regional and national infection control and in implementing surveillance systems that monitor the spread of antimicrobial resistance.

*Infect Control Hosp Epidemiol* 2016;37:761–767

The spread of antimicrobial-resistant microorganisms poses an increasing threat to affordable modern health care.^1^ In the Netherlands, efforts to control the dispersal of known and novel antimicrobial-resistant organisms have been mostly implemented at the hospital level.^2^ However, recent studies have recommended shifting the focus of control strategies from single hospitals toward larger healthcare networks.^3^ ^–^ ^5^ These networks consist of clusters of hospitals that are connected via shared patients. Several studies have shown that patients transferred from one hospital to another can spread antimicrobial-resistant pathogens across the healthcare network.^6^ ^–^ ^11^

Hospitals not only exchange patients with each other but also with other healthcare institutes such as nursing homes.^4^ Although hospitals deliver the most intense, specialized care and treat the most patients per year, nursing homes outnumber them in terms of different locations and estimated number of beds. Whereas in hospitals, rigorous control measures exist for MRSA and other highly antimicrobial-resistant microorganisms,^2^ in nursing homes, guidelines^12^ are less stringent, and compliance with, for example, hand hygiene recommendations is generally low.^13^ ^–^ ^15^ The resulting suboptimal hygiene^16^ in combination with the vulnerable nursing home population and infrequent screening and control measures creates a favorable environment for transmission of antimicrobial-resistant pathogen strains within nursing homes.

Knowledge regarding the spread of antimicrobial-resistant pathogens from nursing homes across the larger healthcare network remains limited, especially in the Netherlands, where levels of antimicrobial-resistant pathogens are still low. In nursing homes, MRSA prevalence is <1%^17^ ^,^ ^18^ and ESBL prevalence is 8%–10% on average (range, 0–20%).^19^ ^,^ ^20^

However, the potential of nursing homes to spread antimicrobial-resistant pathogens has been confirmed by studies in countries with higher levels of these pathogens that have reported a higher prevalence of MRSA in nursing homes than in hospitals^21^ and have identified “being transferred from a long-term care institution” as a risk factor for being carrier of MRSA in patients admitted to the hospital.^22^

In this study, we investigated whether nursing homes should be included in regional and national control strategies for antimicrobial-resistant pathogens. We sought to determine (1) how nursing homes are positioned in the healthcare network and (2) how nursing homes and their connections with hospitals influence the spread of antimicrobial-resistant pathogens across the healthcare network. First, we gathered data regarding patient referrals collected in a nursing home surveillance network. Second, we developed a network model representative of the current Dutch healthcare system to simulate the spread of antimicrobial-resistant pathogens across the network. We compared different scenarios, starting with a model that included only hospitals and the community. We then extended this model to include nursing homes. By comparing different scenarios, we were able to identify the contribution of nursing homes to the spread of antimicrobial-resistant pathogen strains across the healthcare network.

## METHODS

We constructed a synthetic network of hospitals and nursing homes to model the Dutch healthcare system. We assumed that the connections between institutions (nodes) in the network, through which antimicrobial-resistant pathogen strains can spread, were formed exclusively by patient transfers.

### Network Nodes: Nursing Homes and Hospitals

**FIGURE 1:**
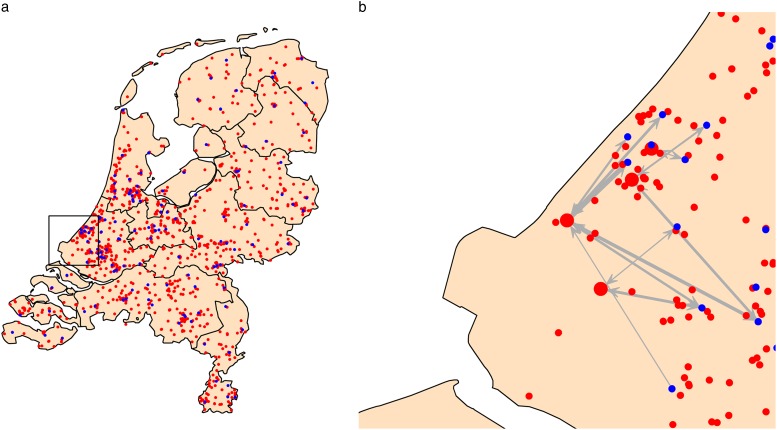
(a) Map of the Netherlands with the locations of hospitals (blue) and nursing homes (red). (b) Example of the yearly simulated patient flow between 4 of the nursing homes (large red dots) and the hospitals (blue).

Early in 2014, we listed all 130 hospital locations in the Netherlands by address and type (ie, academic, top clinical, and regular), excluding specialized care centers^23^ (see Supplementary Information for a description and discussion of possible bias). To identify all nursing homes, we used a list of healthcare organizations from the branch organization Actiz. We searched the website of each of the listed healthcare organizations to find individual long-term care locations. We then evaluated these institutions according to the care they offered and assumed those offering psychogeriatric care, intense somatic care, or rehabilitation care to be a nursing home. In this way, we identified 1,095 separate nursing home locations. These nursing homes and the hospitals comprised the nodes in our network (Figure 1a).

### Network Edges: Patient Transfers

Data pertaining to the flow of patients between nursing homes and hospitals and vice versa were obtained from data collected by the Sentinel Surveillance Network on Infectious Diseases in Nursing Homes (SNIV) in 2012, 2013, and 2014.^24^ Approximately 30 nursing homes had participated that submitted weekly data on the number of patients referred to hospitals and received from hospitals as well as the hospitals concerned during the 3-year study period. We used only data from nursing homes for which we had at least 26 weeks of observations. For each nursing home, also additional data were collected: location, number of beds, and mortality rate.

To evaluate the transfer of patients between hospitals, we used data on the total number of patients moved between each pair of Dutch hospitals in 2004.^8^ Additional data were also collected: locations of the hospitals, the hospital type (ie, academic, top clinical, or regular), and the total number of admissions.

We were unable to collect data for patients transferred from one nursing home to another. Following expert opinion, we assumed these occurrences to be negligible and did not assign any connections between the nursing homes in the model.

### Network Reconstruction and Transition Matrix

We input the available observations regarding patient transfers into our model to reconstruct a complete network representative of our current healthcare system. The main determinants of the patient flow between 2 locations were the distance between them, the type of hospital, and the size of the nursing home involved. A detailed description of the model creation of the complete network including hospitals, nursing homes, and the community (divided into 12 provinces) can be found in the Supplementary Information. There, we have also provided some network statistics for the comparison of our model with the actual network (Supplementary Figure S1). Next, we transformed the rates of patients exchanged per year for hospitals and per week per bed for nursing homes into a transition matrix *P* in which each element *p*_*ij*_ describes the probability per day of a patient in institution *j* to move to institution *i.* The total probabilities in each column *j* of the matrix of either moving or staying (on the diagonal) sum to 1. A detailed description of the calculations of this transition matrix, as well as the parameters used, is provided in the Supplementary Information. Figure 1b shows an example of the simulated patient flow for some of the nursing homes in the network.

### Colonization and Transmission

Every node in the network (ie, hospital, nursing home, and province) was considered a separate population. If a novel antimicrobial-resistant pathogen strain emerged in one of the populations, we assumed that it spread across the network via colonized patients that moved from one population to another. We assumed that being colonized did not impact the length of stay and the probability of being transferred.

Within a population, a pathogen can be transmitted from a colonized individual to susceptible individuals according to the susceptible–infected (ie, colonized)–susceptible (SIS) model.^25^ We assumed that patients mixed homogeneously such that everyone could transmit to everyone within a population. Also, for this study, we assumed that no transmission occurred in the community outside the healthcare institutions. In each institution, a colonized patient could cause new colonizations at a rate of β*S*/*N* per day in which *S* is the number of susceptible individuals in the population, *N* is the total number of individuals in the population, and β is the transmissibility. The transmissibility combines the probability of contact between individuals and the transmission probability per contact.

For each simulation, we chose one value for the transmission parameters β_*h*_ and β_*n*_ for hospitals and nursing homes respectively, and we used different values in different simulations. In our model, an individual could lose colonization at a rate μ, which we chose to be 1/187.5 per day, such that the average duration of colonization was τ=0.5 year, which is in line with observations of long-term carriage of resistant strains (eg, MRSA and ESBL).^26^ ^–^ ^28^ From the parameters β and μ, we calculated 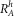 and 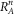, the basic reproduction numbers for a single admission episode in hospitals and nursing homes, respectively.^29^ This admission reproduction number is defined as the average number of new colonizations caused by 1 colonized individual in an entirely susceptible population during 1 admission episode. *R*_*A*_ can be calculated as the product of the transmissibility β_*h*_ or β_*n*_ and the duration that a colonized patient can infect others. In nursing homes in our simulation, the average length of stay was longer than the duration of colonization; thus, the time frame in which others could be colonized depended mainly on the duration of colonization (τ) and 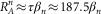. For hospitals, the average length of stay was much shorter than the average duration of colonization; thus, 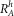 was mainly determined by the length of stay, and 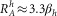. Therefore, in our model, if we assumed the transmissibility β to be the same in hospitals and nursing homes, by definition, 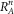 was >50 times larger than 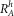.

Simulations for this scenario for realistic values of 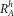 (≥1) showed enormous outbreaks in nursing homes, hospitals, and the general population. Given the low prevalence and rarity of large outbreaks in nursing homes in the Netherlands, we assumed the transmissibility in nursing homes to be lower than in hospitals, and we ran the model for values of β_*n*_ chosen such that 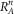 was <1 or >1.

### Simulation

Each simulation describes a period of 10 years with time steps of 1 day, starting with a small outbreak of 10 cases in 1 randomly chosen hospital. We performed simulations for varying values of β_*h*_ and β_*n*_. For each scenario, we performed 1,600 simulations with 1,600 stochastically created transition matrices. In the first scenarios, we used a network that consisted of hospitals only (and provinces). In the following scenarios, we used the complete network, as described above, including nursing homes.

## RESULTS

Model simulations with a network that contained only hospitals showed that sustained outbreaks occurred only if 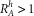. In all simulations with 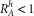, extinction occurred (Supplementary Figure S2). The final endemic prevalence and the growth rate of the epidemic depended on the value of 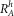. The average prevalence across all hospitals was more or less stable after the initial phase of a few years, but the prevalence within individual hospitals fluctuated (Supplementary Figure S3).

To study the impact of nursing homes on the spread of new nosocomial pathogens across the healthcare network, we then performed simulations with the complete network, including both hospitals and nursing homes for scenarios in which 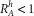 (ie, 0.81) and 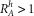 (ie, 1.13).

**FIGURE 2:**
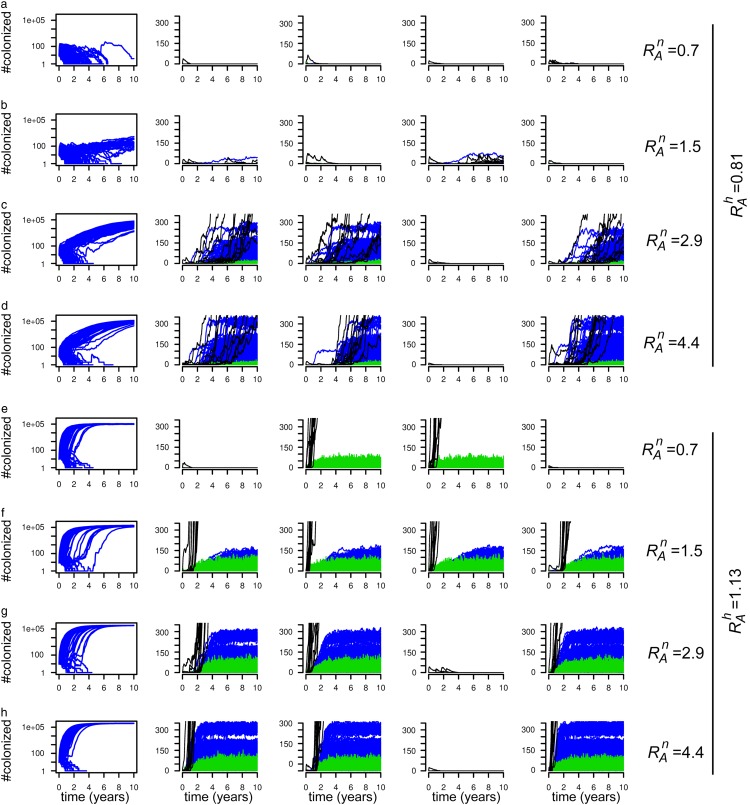
Prevalence of colonization in simulations on the complete hospital-nursing home network, for 8 different combinations of the transmissibility parameters: (a) β_*h*_=0.25 and β_*n*_=0.005, (b) β_*h*_=0.25 and β_*n*_=0.01, (c) β_*h*_=0.25 and β_*n*_=0.02, (d) β_*h*_=0.25 and β*n*=0.03, (e) β_*h*_=0.35 and β_*n*_=0.005, (f) β_*h*_=0.35 and β_*n*_=0.01, (g) β_*h*_=0.35 and β_*n*_=0.02, (h) β_*h*_=0.35 and β_*n*_=0.03. The graphs in the first column show the total prevalence of colonization over time for 100 different simulations. The other four graphs show the result of individual simulations. Each line shows the prevalence in either a hospital (green), a nursing home (blue) or the community (per province, black).

In scenarios in which 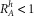, no sustained outbreaks occurred through hospital transmission alone (Figure S2). However, if transmissibility in nursing homes, β_*n*_ increased such that 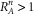, the initial outbreak could lead to endemicity (Figure 2a–d). In the endemic situation, colonized individuals were present not only in nursing homes but via patient referrals and discharge also in hospitals and the community. Individual runs for these scenarios (Figure 2a–d, right 4 columns) showed that the initial outbreak either died out after all colonized patients were discharged from the hospital or it increased after colonization was introduced into 1 or more nursing homes, from which it spread to the community and hospitals. The growth rate of the epidemic and the final endemic prevalence depended on the values of both 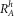 and 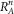 (Figure 2, left column). The final prevalence in nursing homes and the impact on hospitals depended on the value of 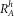. In the fourth scenario, where 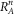 was 4.4, the average hospital prevalence grew to 3% after 10 years.

In the scenarios with 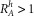 (Figure 2e–h), increased transmission in nursing homes was no longer necessary for a sustained outbreak, but it increased the total number of patients, not only in nursing homes, but also in hospitals. For the chosen parameter values, the average hospital prevalence after 10 years was 18% when 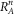 was 0.7 (Figure 2e) and 30% when 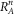 was 4.4 (Figure 2h).

## DISCUSSION

Our modeling study shows that nursing homes in the Netherlands are sufficiently connected to the hospital network to drive national epidemics. If a new pathogen emerges that spreads easily in the nursing home environment, the large number of nursing homes, with their connections to hospitals and the community, can, in the absence of control measures, sustain or initiate nationwide outbreaks. The long average length of stay of patients in nursing homes creates a long window of opportunity for transmission, and the threshold value of the daily transmissibility for outbreaks to occur is much lower than in hospitals. If the basic reproductive number for a single admission episode is >1(in either hospitals or nursing homes), sustained transmission can occur, followed by large outbreaks.

We are not the first to model the transmission of pathogens between and from long-term care facilities.^30^ ^–^ ^32^ In another modeling study, Lee et al^32^ demonstrated that nursing homes are important in the spread of MRSA among hospitals in California, where MRSA is endemic. Their results suggested that in designing control efforts, hospitals should keep track of how they are connected to nursing homes via shared patients. Our study adds to this idea by showing that nursing homes might also be important for infection control in earlier stages after the emergence of a new pathogen, when endemicity has not yet been reached. Our findings are also supported by a study by the CDC showing that a coordinated approach by interconnected healthcare institutes to interrupting transmission of resistant pathogens is more effective than historically independent facility-based efforts.^33^

In this study, as in most modeling studies, we have made a few assumptions that require further discussion. First, we assumed that links between healthcare institutions are exclusively formed by transferred patients. In reality, a fraction of healthcare workers, especially flex and temporary workers and students, also tend to work in 1 or more healthcare institutions at the same time or within a short time frame. Taking this into account would make the network even more connected and would reflect an increase in the potential for spread of pathogens between facilities.

Second, we assumed homogeneous mixing of the population within each institution. In reality, not all individuals in a healthcare institution have the same probability of meeting each other and ideally, true contact patterns would be incorporated in the model. However, we relied on previous work showing that as long as there are at least some random contacts in reality, the network dynamics of a simulation model with random mixing perform similarly to those including more detailed contact patterns.^34^

A third assumption involves the transmissibility parameters β_*n*_ and β_*h*_, which we assumed to be the same for all nursing homes and hospitals, respectively. Actually, large differences exist between the institutions concerning patient population, hygiene, staff–patient ratios, contact patterns and contact precautions. All of these factors influence the transmission of pathogens. In the future, more elaborate models will benefit from the collection of more detailed data on these topics.

Finally and importantly, we assumed that all colonized individuals remained unnoticed and that no control measures were applied. Although this is mostly true during the beginning of an outbreak, at some point, usually the hospital staff becomes aware of an emerging pathogen and control measures are introduced. In nursing homes, with less intensive screening and with less carriers developing an infection, transmission can remain unnoticed much longer. Our model simulations show that when transmission in hospitals is insufficient to generate large outbreaks, nursing homes can play a crucial role in facilitating sustained transmission across the healthcare network. We believe our results are valid not only for pathogens with low natural transmission potential in hospitals but also for situations in which control measures reduce transmission. Such is the case for MRSA in hospitals in the Netherlands where the search and control policy is in place.

Our study reveals that nursing homes have a high potential for spread of new pathogenic strains across a healthcare network. If the daily probability of transmission of colonization was the same in hospitals and nursing homes, we would expect a much higher prevalence of colonization in nursing homes than in hospitals because of the longer length of stay creating a longer opportunity for transmission. The fact that prevalence of colonization with MRSA is still very low in nursing homes in our country^17^ suggests that, despite the difference in infection prevention measures between hospitals and nursing homes, the daily probability of transmission of MRSA is lower in nursing homes than in hospitals. This characteristic might be strain dependent, a hypothesis that is supported by a recent outbreak of MRSA t1081 that occurred predominantly in nursing homes.^35^

Our study suggests that negative surveillance data, which are often based on clinical infections and usually do not cover the entire healthcare system, should be interpreted with care and should not lead us to conclude prematurely that the healthcare network is well protected against outbreaks. Few or no cases detected at a certain point in time might indicate that no outbreak is occurring, but such a period might also be the initial phase after the introduction of a new pathogen, in which prevalence is increasing in only 1 or a few institutions before being transferred to other places. The initial process might take 1 year or a few years, but if uninterrupted, it will eventually lead to a large outbreak.

In summary, if a pathogen emerges that is easily transmitted within nursing homes, control efforts aimed at hospitals might no longer be effective in preventing nationwide outbreaks. As the level of transmissibility required for sustained outbreaks in nursing homes is an order of magnitude lower than in hospitals, the probability of emergence of a pathogen that can cause nursing home outbreaks is considerable. Therefore, not only nursing home infection control should be improved, but nursing homes should be considered in planning for regional and national infection control and surveillance initiatives.

## Supplementary material

For supplementary material accompanying this paper visit http://dx.doi.org/10.1017/ice.2016.59. click here to view supplementary material
