# Supplementary material for: The Role of Nursing Homes in the Spread of Antimicrobial Resistance Over the Healthcare Network
Source: Infect Control Hosp Epidemiol. 2016 Apr 7;37(7):761–7. doi: 10.1017/ice.2016.59 (PMC4926272; doi:10.1017/ice.2016.59)
Supplement: Supplementary file 1 [file S0899823X16000593sup001.docx]

## Supplementary Information

## Exclusion of specialized care centers

The main reason for excluding specialized care centers from our model is that we do not have data of patient referrals from most of these centers. The patient referral data that were used in this study were sent in by general, topclinical and academic hospitals (98 in total in 2004). In 2013 there were 82 of these hospitals with in total 131 locations. For comparison, in 2015 there were 22 specialized care centers (for eg epilepsy, asthma, cancer, audiology, radiotherapeutics or dialysis), some of them with several locations. The distributions of these centers over the Netherlands is similar to the distribution of general hospitals, with higher numbers in more densely populated areas. Ideally we would like to include information for all centers offering patient care, but because of lack of these data we have decided to restrict our model to the general hospitals, as was also done in previous studies on this topic. Another reason for excluding them is the fact that these specialized care centers are very restricted in terms of patient population and might not be easily compared with general hospitals. We think that exclusion of these centers does not influence the results of this study as the number of patients treated is smaller than in the included hospitals and patient exchange with nursing homes is probably also low.

## From available data to network construction

To estimate the patient flow from nursing home to hospital and vice versa, we randomly choose an actual reference nursing home from the SNIV (Sentinel Surveillance Network on Infectious diseases in nursing homes) dataset for each nursing home in our simulated network. We assume the total in- or outflow (number of patients moved per day per bed) of the simulated nursing home to be the same as that of the reference nursing home. We distinguish between the 3 types of hospitals (academic, top clinical and regular) and for each type we rank the actual hospitals according to their distance to the reference nursing home. For the simulated nursing home we also rank the simulated hospitals per type according to their distance to the simulated nursing home. Then we copy the flow between the reference nursing home and its first ranked actual hospital to the simulated nursing home and its first ranked simulated hospital, and so on for the lower ranked hospitals.

For estimation of the patient flow between hospitals, we use a similar strategy. We randomly choose a reference actual hospital of the same type as the simulated hospital and rank the other hospitals based on their geographic distance to the reference or simulated hospital. Since in this case the number of hospitals in the observed network is not the same as the one in the simulated network (numbers have changed since 2004), we cannot directly copy patient flows by ranking, but instead distribute them evenly. For example, if the observed network contains 7 hospitals and the simulated 10, then the first simulated hospital gets 7/10 of the flow from the first ranked observed hospital, the second, gets 3/10 of the flow from the first ranked observed hospital and 4/10 from the second ranked observed hospital and so on. In this way we copy both inflow and outflow data for the simulated network. If we do so for each hospital, we obtain two sets of data for each couple of hospitals in the simulated network (one based on each hospital in the couple). Since we want inflow and outflow between a pair of hospitals to have the same correlation as we observe in the actual data, for each set of hospitals we randomly choose either of the two sets of flow data.

**Network comparison**

To check how well our synthetic network approximates the actual network, we compare some network statistics. Since we have most complete data on hospital-hospital transfers, we choose to compare the between hospital networks. We find that the synthetic network is similar in the sense that the average number of connections of the nodes is close to the one in the original network, as well as the indegree (i.e. the annual number of patients referred to each hospital from the other hospitals) and outdegree (the number of patients referred from each hospital to the other hospitals). In other, more complex, network descriptive statistics such as closeness and betweenness we find some differences (See figure S1).

**Building of the transition matrix**

To construct a transition matrix, we transform the available data on the rates of patients exchanged per year for hospitals and per week per bed for nursing homes into a matrix *P* in which each element *p_ij_* describes the probability per day of a patient in institution *j* to move to institution *i.* To this end, we first divide each column *j* of the flow matrix by the number of beds in institution *j*, such that we obtain a rate per patient (*r_ij_*). The number of beds in each hospital (i.e. the average number of occupied beds per day) is calculated from the total number of admissions to each hospital and the average length of stay over all hospitals. Both based on the annual report Care 2013 from the Ministry of Health, Welfare and Sport.[^1^](#_ENREF_1) In absence of data, the number of beds in each nursing home is drawn randomly from the observed size distribution of nursing homes participating in SNIV.

To calculate the probability that a patient moves from institution *j* to i, we use $p_{ij}=1-e^{-r_{ij}t}$, in which we take *t*=1 day.

We add 12 nodes, each of these representing the general population in one of the 12 provinces in the Netherlands. We introduce a ‘source’ node to account for demographic processes (birth/death). We assume individuals are only admitted from and discharged to the community in the province in which the hospital or nursing home is located. We estimate the probability for someone in the hospital to be discharged each week from the average length of stay of 3.3 days (estimated from the annual report Care 2013 [^1^](#_ENREF_1)), $p_{D}=1-e^{-\left( \frac{1}{3.3} \right)}$. The probability that a person in institution *j* is discharged to the community in its province (*z*) is the probability of discharge, minus the probability of death ($2\%$of the hospital patients[^2^](#_ENREF_2)) and minus the probability of moving to another institution: $p_{zj}$=$p_{D}-p_{m}-\sum_{i\neq z,j} pij$.

Since in our model the number of patients (= the number of occupied beds) in an institution is assumed to be stable during the simulated period, the probability for someone in province *z* to be admitted to institution *i, p_iz_* is equal to the sum of all probabilities of discharge (ie the probability/fraction of empty beds), times the number of beds in institution *i, b_i,_* divided by the population size of the province (*N­_p_*): $p_{iz}$=$1-e^{-\frac{b_{i}}{N_{p}}\times(1/4.3)}$.

In the SNIV study the average mortality rate was 9.2x10^-4^ per patient per day, thus the probability for a nursing home patient to die is $p_{mj}=(1-e^{-0.00092})$. No data were collected on the length of stay or total numbers of admitted or discharged patients in the nursing homes. However, according to a study by Ribbe et al[^3^](#_ENREF_3) on average 1 in 3 nursing home patients recovers and is discharged, while the others die. If we take the average discharge rate to be the half of the mortality rate, we expect an average length of stay of 728 days. Thus the daily probability for a patient in nursing home *j* to be discharged to the community (province *z*) is the total probability of discharge minus the probability to be discharged to a hospital, $p_{zj}=\left( 1-e^{-0.00046} \right)-\sum_{i\neq z} pij$. Since we assume each nursing home bed is filled immediately after discharge or death of a patient (ie the number of patients is constant), the rate of admission has to be equal to the rate of discharge plus death. The probability of someone in society to be admitted to the nursing home *i*, thus is equal to the probability of an empty bed minus the probability that the bed is filled with patients from a hospital: $p_{iz}=(1-e^{-0.00137\times\frac{B_{i}}{N_{p}}})$-$\sum_{j\neq z} pij\times B_{j}/B_{i}$.

For some nursing homes, the patient admission rate from the hospitals alone is larger than the total admission rate calculated from the average length of stay. For these nursing homes we suppose that the number of admissions from the community is negligible compared to that from the hospitals and we adapt the rate of discharge to the higher rate of admission.

The mortality rate In the community is set to 1/(80*365) per day, such that the average life expectancy is 80 years. To maintain a constant population size, we assume the daily number of births is equal to the number of deaths. We assume 70% of the births to take place in the hospital and 30% at home.[^4^](#_ENREF_4) For each province we distribute the births over the hospitals proportionally to the number of hospital beds.

Individuals in the community are only admitted to institutions in their own province. We do however allow individuals in the community to move between provinces. The probabilities of moving from one province to another are calculated from data from Statistics Netherlands.[^5^](#_ENREF_5)

On the diagonal of the matrix we calculate the probability of staying in the same institution, which is $p_{jj}=1-\sum_{i\neq j} p_{ij}$.

## Supplementary figures


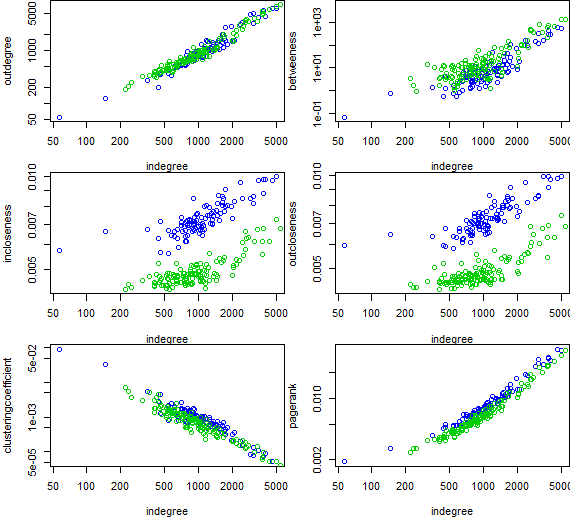


**Figure S1.** Comparison of network statistics for the observed hospital network (blue) and a simulated hospital network (green). Each hospital is a node in the network. The outdegree describes the total number of patients received from other hospitals per year. The indegree describes the total number of patients referred to other hospitals per year. Incloseness and outcloseness are defined as the inverse of the average length of the shortest paths to or from all the other nodes in the graph respectively. The clustering coefficient measures the probability that the neighbouring nodes of a given node are also directly connected. The pagerank gives the Google pagerank for all nodes.[^6^](#_ENREF_6).


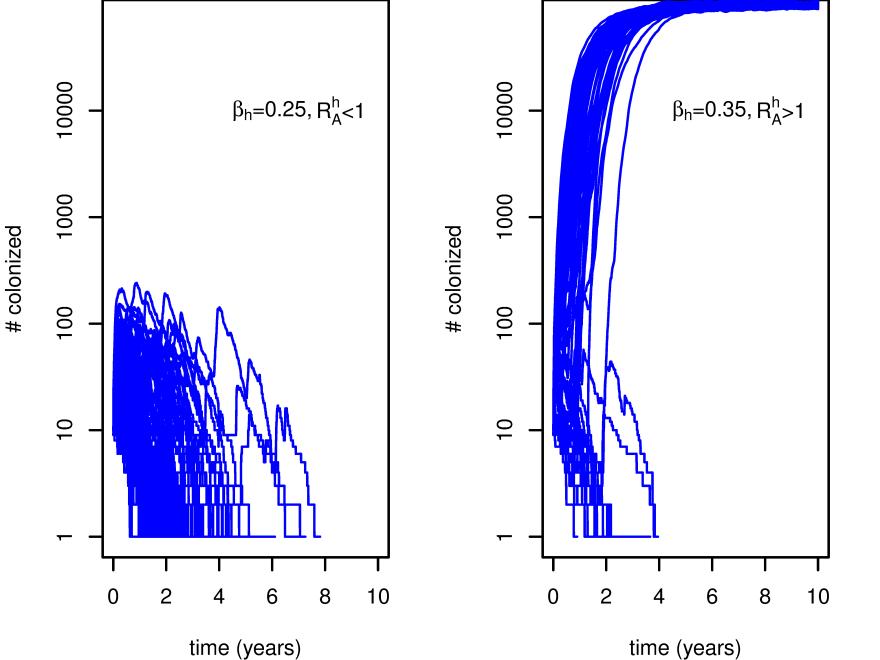


**Figure S2.** Stochastic simulations of the reconstructed Dutch hospital network for different values of the transmission parameter $\beta_{h}$ and resulting difference in $R_{A}^{h}$(0.81 and 1.13 respectively).

**
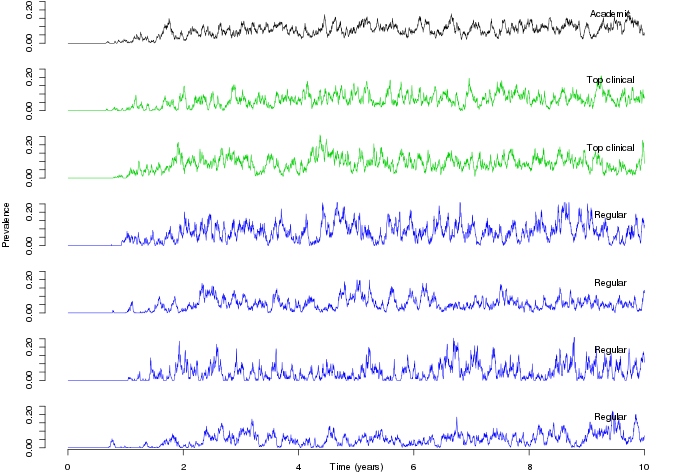
**

**Figure S3.** Results for one simulation for the hospital-only network. $\beta_{h}$ is 0.35 and $R_{0}^{h}$ is 1.13. Shown are prevalence over time in one academic hospital, two top clinical hospitals and four general hospitals, all randomly chosen.

**References**

1. Jaarverslagen zorg. Ministerie van Volksgezondheid WeS. 2013. https://[www.jaarverslagenzorg.nl/zorginstellingen/jaarverantwoordingzorgwieenwaarom/gebruikgegevens/openbaredatasetsdigimv.asp](http://www.jaarverslagenzorg.nl/zorginstellingen/jaarverantwoordingzorgwieenwaarom/gebruikgegevens/openbaredatasetsdigimv.asp).

2. Hall MJ, Levant S, DeFrances CJ. Trends in inpatient hospital deaths: National Hospital Discharge Survey, 2000-2010. *NCHS Data Brief* 2013:1-8.

3. Ribbe MW, van Mens JT, Frijters DH. [Characteristics of patients during their stay in a nursing home and at discharge]. *Ned Tijdschr Geneeskd* 1995;139:123-127.

4. RIVM. Geboortezorg in Nederland. 2014.

5. Tussen gemeenten verhuisde personen. Statistics Netherlands. 2013. <http://statline.cbs.nl/Statweb/>.

6. The Anatomy of a Large-Scale Hypertextual Web Search Engine. Brin S, Page L. <http://infolab.stanford.edu/~backrub/google.html>.
